# Combined Assessment of the Obstetrical Conjugate and Fetal Birth Weight Predicts Birth Mode Outcome in Vaginally Intended Breech Deliveries of Primiparous Women—A Frabat Study

**DOI:** 10.3390/jcm11113201

**Published:** 2022-06-03

**Authors:** Nadja Zander, Florian J. Raimann, Ammar Al Naimi, Dörthe Brüggmann, Frank Louwen, Lukas Jennewein

**Affiliations:** 1 Department of Midwifery Frankfurt, Goethe University, 60590 Frankfurt, Germany; nadja.zander@web.de; 2 Department of Anaesthesiology, Intensive Care Medicine and Pain Therapy, University Hospital Frankfurt, Goethe University, 60590 Frankfurt, Germany; florian.raimann@kgu.de; 3 Department of Gynecology and Obstetrics, School of Medicine, Goethe University, Theodor-Stern-Kai 7, 60590 Frankfurt, Germany; ammar.alnaimi@kgu.de (A.A.N.); doerthe.brueggmann@kgu.de (D.B.); louwen@em.uni-frankfurt.de (F.L.)

**Keywords:** breech, delivery mode, obstetrical conjugate, birth weight

## Abstract

(1) Background: Guidelines on vaginal breech delivery require birth weight restrictions and neglect the impact of pelvic measurements despite contradicting evidence. There is a great need for more evidence on delivery outcome predicting factors for patients counselling. (2) Methods: We performed a prospective cohort study on 748 primiparous women intending vaginal breech birth and analyzed combined influence of fetal birth weight (BW) and the obstetric conjugate (conjugate vera obstetrica, CVO) on delivery outcome. (3) Results: We generated a BW/CVO ratio and devided our study cohort at median (257.8 g/cm) into a low ratio group (LR, with low birth weight and wide obstetric conjugate) and a high ratio group (HR, high birth weight and narrow obstetric conjugate). Cesarean section (CS) rate was significantly higher in HR (50.3%) as compared to LR (28.3%, *p* < 0.0001). Fetal morbidity was not different. In vaginally completed deliveries duration of birth was significantly longer in vHR (557 min) as in vLR (414 min, *p* < 0.001). Manual assistance to deliver the arms (‘Louwen maneuver’) positively correlated with birth weight (r^2^ = 0.215; *p* = 0.005) and the BW/CVO ratio (r^2^ = 0.0147; *p* = 0.02). (4) Conclusions: A high fetal birth weight combined with a tiny CVO predicts higher cesarean section probability, longer birth duration and the necessity to perform arm delivery assistance. Birth weight and pelvic measurements should be topics of great importance in patients counselling.

## 1. Introduction

Vaginal delivery of fetuses in breech presentation has been established as a safe delivery option during the last decade. Numerous studies have shown that with careful patient selection, an experienced obstetrical team and an upright birth position maternal and fetal long-term outcome is not of disadvantage, compared to an elective cesarean section [1,2,3,4,5,6]. Accordingly, national guidelines support the vaginal birth approach [7,8,9]. These guidelines limit the recommendation for a vaginally attempted birth with a fetal birth weight to 3800 g or 4000 g. They also describe the role of pelvimetric measurements as unclear. Opposingly, data of the biggest monocentric study cohort on vaginally intended breech deliveries shows that an upper birth weight limit is not necessary [10] and a correlation between the birth mode and pelvic measurements is recognizable [11,12].

In counselling, patients have to decide on their attempted delivery mode based on the expertise and the information given by their obstetricians. Evidence based shared decision making should be the goal in each counseling session in order to succeed in finding the best individual approach for each patient. This includes explaining possible complications of an elective cesarean section, namely postpartum bleeding, wound infection and subsequent pregnancies complicated by previous cesarean section. In this context, avoiding a cesarean section should be discussed addressing fetal and maternal outcome as well as the probability to receive a cesarean section after the onset of labor. Of highest importance is safety in terms of fetal and maternal morbidity. Louwen et al. showed that fetal morbidity can be reduced when women deliver in an upright position [6]. Also, individual risk factors leading to a higher probability for an emergency cesarean section have to be considered. This important issue often is left out in counselling because reliable data is scarce. Jennewein et al. showed, that cesarean section probability rises with increasing fetal birth weight without an impact on feto-maternal outcome in vaginally attempted breech deliveries [10]. Primiparous women, women who already had a cesarean before and women who pass their due date are more likely to have a cesarean section during birth [13,14,15,16]. All mentioned factors do not impact fetal morbidity rates. In counselling, the probability of a shoulder dystocia and a delayed head delivery have to be addressed, too. Both complications easily can be managed by obstetricians when the mother giving birth is in an upright position. The ‘Louwen maneuver” to assist arm delivery in a breech shoulder dystocia and the ‘Frank nudge’ to help with head delivery if delayed, described by Louwen et al. [6] easily can be learned and implemented [17]. An obstetrical conjugate (conjugata vera obstetrica, CVO) lower than 12 cm is reported to be an important risk factor for a cesarean section [11,18,19]. To date, over all data on pelvic measurements and its predictive value on birth outcome is not enough in order to be implemented into guideline recommendations [7,8,20]. Since pelvic characteristics and the fetal birth weight together comprehensibly are correlating with vaginal or cesarean birth, we conducted a prospective cohort study on 748 primiparous women analyzing fetal birth weight and the CVO with their combined impact on delivery outcome in vaginally intended breech birth.

## 2. Materials and Methods

### 2.1. Patient Cohort and Patient Selection

The study was performed in accordance to the actual Helsinki Declaration. We performed a prospective cohort study on vaginally intended deliveries of term singletons in breech presentation (>37 weeks of pregnancy) of primiparous women at the Goethe University Hospital in Frankfurt from 01/2004–12/2019. Patients with complications with an impact on delivery mode, deliveries with a fetal birth weight of beneath 2500 g, multiple pregnancies and patients with not sufficiently treated diabetic illnesses were excluded from the study. As a standard in out center, all women with a breech presentation come for counseling prior to birth in which the benefits and risks of vaginally intended birth as well as planned cesarean section are explained. The university clinic’s ethics committee gave consent (420/11). Informed written consent was waived because data was mainly gathered after patient’s dismission. Standard clinical care applied. Vaginal breech deliveries are performed predominantly in an upright birth position. In rare cases, by choice of the patient or the obstetrician in charge, deliveries were continued/ended in dorsal position. Our analyses of vaginal deliveries exclude these births (*n* = 88) in this study because the birth position has been shown to influence birth outcome [5]. An upright position is defined as a birth position of the mother either on hands and knees, in a squatting position, kneeling or in a standing position. In our center, most women gave birth on hands and knees when they delivered vaginally.

### 2.2. Data Collection

The state database ’Perinatalerhebung Hessen’ and the hospital’s patient management system was utilized for data acquisition. CVO values were measured with MRI imaging between 35–38 completed weeks of gestation by licensed radiologists and quality checked by experienced obstetricians. All primiparous women receive an MRI of the pelvis during counselling. The obstetric conjugate (CVO) is defined as the distance from the promontory to the back of the symphysis pubis.

Counselling process, cohort selection and the hospitals regiments on vaginal breech delivery have been previously described within FRABAT study cohort publications [6,10,13,14,15,21].

### 2.3. Data Preparation

We generated two main study cohort subgroups. In order to do that we generated a birth weight/obstetric conjugate (CVO) ratio (BW/CVO). The study group was then divided by median split. The median of the whole cohort’s birth weight/CVO ratio was 257.8 g/cm. The division resulted in two study groups: One with a low birth weight and large obstetric conjugate (low ratio, LR) and one group with high birth weight and a small obstetric conjugate (high ratio, HR). The latter being the group with expected less favorable outcomes. Median split was performed in order to get two groups with equal sample size The modified PREMODA score used in this study previously has been described in other publications of the FRABAT collective [6,10,13,14,15,21]. A case counts as a delivery with fetal short term morbidity if one or more of the following items apply: intubation period > 24 h, stay on the neonatal intensive care unit of over 4 days, neurological deficit, 5 min. APGAR value < 4, fetal birth injury.

### 2.4. Statistical Analyses

Variables were tested if normal distribution applied with the Kolmogorov-Smirnov test. Pearson’s χ^2^-test was used to detect group differences. Statistical analyses were performed using JMP software (Version 14.0, SAS Institute, Cary, NC, USA). A *p*-value of below 0.05 was considered as statistically significant.

## 3. Results

**Figure 1:**
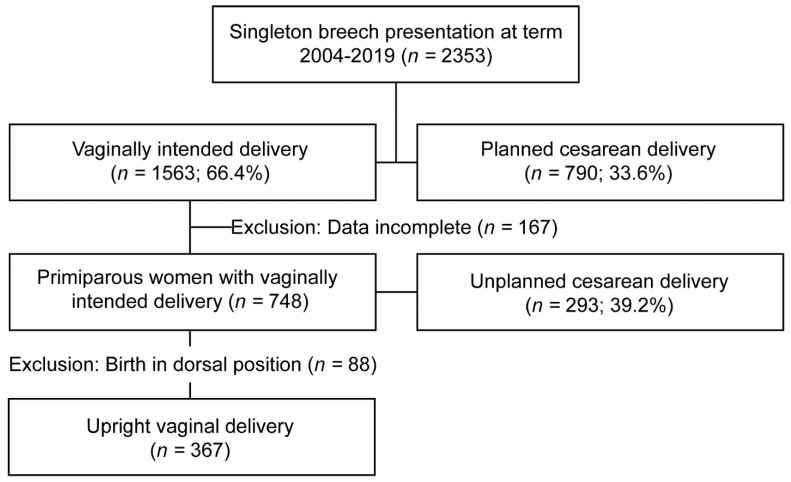
Flow Chart Study cohort.

2353 patients were counselled regarding their delivery out of breech presentation from 2004–2019 with delivery ≥37 weeks of gestation. 1563 mothers opted for a vaginal attempt of whom 915 patients were primiparaous. Patients who opted for planned cesarean had medical indication for cesarean delivery independent from the fetal presentation or decided to deliver by cesarean section. We had to exclude 167 patients due to incomplete data (Figure 1). 758 cases were included in our analysis. Mean age was 31.3 years, mean BMI was 23.0 kg/m^2^. 100 women had minor preconditions (e.g., hypothyroidism, hypertension, diabetes). Mean birth weight was 3339 g, mean obstetric conjugate (conjugate vera obstetrica, CVO) was 12.9 cm and overall cesarean section rate was 39.2% (Supplementary Table S1).

We hypothesized a beneficial impact of a larger CVO and a disadvantegous influence of great fetal birth weight on delivery outcome parameters. In order to detect the combined impact of fetal birth weight (BW) and the CVO, we generated a Birth weight/CVO ratio. We performed a median split (Median of Birth weight/CVO Ratio = 257.8 g/cm) in order to generate two groups with equal sample sizes in a low ratio group (LR) and a high ratio group (HR).

In the cohort of vaginally intended deliveries, pregnant women in HR had higher BMI values (LR: 22.6 kg/m^2^, HR: 23.3 kg/m^2^; *p* = 0.001) and a longer duration of pregnancy (LR: 277 days; HR: 282 days; *p* < 0.0001; Table 1). Maternal precondition rates were equally distributed. Mean birth weight in LR was 3065 g, in HR 3613 g (Table 1). Mean CVO was 13.1 cm in LR and 12.7 cm in HR. Cesarean section (CS) rate was significantly higher in HR (28.3% in LR vs. 50.3% in HR, *p* < 0.001). Significantly more patients received an epidural anesthesia in HR (69.3%) compared to LR (62.0%; *p* = 0.038, Table 1). Fetal short morbidity characteristics did not differ significantly between groups (Table 1). Especially the combined PREMODA score possibly related to birth mode was not significantly different between LR (2.1%) and HR (3.5%; *p* = 0.268; Table 1).

**Figure 2:**
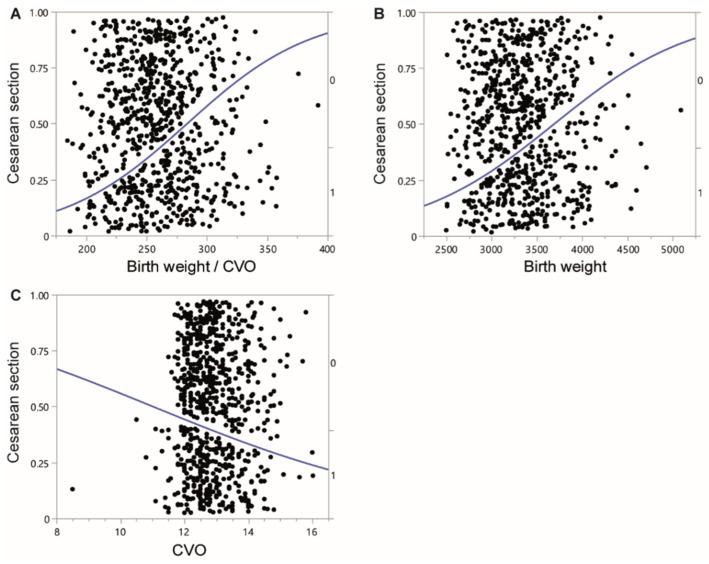
Linear regression of CS rate and (**A**) BW/CVO ratio, (**B**) Birth weight in gram and (**C**) CVO in cm in vaginally intended breech deliveries.

A logistic regression analysis of all vaginal intended births showed a significant positive correlation between BW and the BW/CVO ratio with CS rate. There were significant positive correlations of CS rate and both birth weight and BW/CVO ratio (Figure 2A,B and Table 2) and a significant negative correlation of CS rate and CVO (Figure 2C, Table 2). Inverted prediction revealed, that a BW/CVO-ratio of above 283.8 g/cm predicts a CS rate of above 50% (Table 2). We analyzed BW and CVO independently from its ratio. A BW of over 3691 g predicts a CS rate of over 50%. A prediction of a CS rate of above 50% was calculated with a CVO of below 11 cm (Table 2).

To depict, if CS indications were differently distributed between groups, we performed a subgroup analysis of all CS cases, comparing the low ratio group (cLR) with the high ratio group (cHR). Most CS indications, e.g., birth arrest in first or second stage, mother’s wish or suspected amniotic infection were equally distributed (Table 3). There was a significantly higher amount of CS due to non-reassuring fetal heart rate in cLR with 38.7% compared to 21.3% in cHR (*p* = 0.001; Table 3). Feto-pelvical disproportion as a reason to perform a CS was significantly more often in cHR (9.0%) as in cLR (1.9%; *p* = 0.017; Table 3).

In order to elucidate the impact of CVO and BW on the necessity to perform manual assistance in vaginal deliveries, we analyzed the sub cohorts of vaginal births in HR (vHR) and LR (vLR) after excluding births in dorsal position. There were significantly more births with an epidural anesthesia in vHR (67.6%) as compared to vLR (56.9%; *p* = 0.040; Table 4). Duration of birth was significantly longer in vHR (557 min) as in vLR (414 min; *p* = 0.001; Table 4). Necessity to perform manual assistance was not significantly higher in vHR (49.3%) as compared to vLR (41.7%; *p* = 0.152; Table 5). Fetal morbidity was not significantly different between groups (vLR: 0.9%, vHR: 3.4%; *p* = 0.092; Table 4). Perineal injuries did not occur significantly more often in either group (vLR: 58.6%, vHR: 54.9%; *p* = 0.482; Table 4).

With a logistic regression analysis addressing all vaginal deliveries in an upright position we found that there was no significant correlation of the total amount of manual assistance with either BW, CVO or BW/CVO ratio. There was a significant positive correlation of BW and assisted head delivery (r^2^ = 0.0056; *p* = 0.095; Table 5). There also was a significant positive correlation of the BW/CVO ratio and assisted arm delivery (r^2^ = 0.015; *p* = 0.020; Table 5) and birth weight and assisted arm delivery (r^2^ = 0.022; *p* = 0.005; Table 5).

## 4. Discussion

Vaginal breech birth in an upright position meanwhile is an established and safe birth mode [3,4,8,22]. In guidelines, birth weight restrictions apply and the role of pelvimetric measurements is stated as unclear [7,8,23]. Clinical management of breech presentation is evolving, considering the role of pelvic measurements [24]. Hoffmann et al. document the importance of pelvic measurement in breech birth counseling [12]. More data enabling birth mode and delivery outcome prediction is necessary in order to improve patients counselling. Because a combined impact of the CVO and fetal birth weight seems obvious, we here investigated the impact of both parameters in combination on delivery outcome for the first time in primiparous women with vaginal birth attempt.

We used a ratio of fetal birth weight and the CVO to create two sub cohorts with equal sample size by median splitting. In the low ratio group (LR), where favorable birth outcome was hypothesized mean birth weight was 3065 g and mean CVO was 13.1 cm. In the high ratio group (HR), mean birth weight was 3613 g and mean CVO was 12.7 cm. Fetal morbidity measured with a modified PREMODA score, adapted from the PREMODA study [4] was not significantly different between groups (Table 1), supporting the data of several studies emphasizing a connection between pelvic measurement and birth outcome [10,11,18,19]. The cesarean section (CS) rate differed significantly with 28.3% (LR) versus 50.3% (HR, Table 1). This clearly shows that an unfavorable proportion of fetal birth weight and the CVO is predictive for the necessity to perform a cesarean section. Looking at the reasons for CS in both groups is even more insightful. Significantly more CS were performed due to feto-maternal disproportion in the HR group (Table 3). In logistic regression analyzes we were able to show, that fetal birth weight, the CVO and the BW/CVO ratio significantly correlate with CS (Figure 2, Table 2). Inverted correlations show that a birth weight of above 3691 g, a CVO of beneath 11 cm or a birth weight/CVO ratio of above 383.8 g/cm result in a probability of over 50% to receive a cesarean section. These values might be used in patients counseling when odds for a successful vaginal birth are discussed. Of course, the inaccuracy of fetal weight estimation has to be noted. In breech as in vertex presentation the inaccuracy is estimated to be 5–10% [25,26]. Because fetal morbidity is not associated with a high birth weight/CVO ratio, cesarean section indications do not arise. Patients should be able to decide on their birth mode with as much information as possible. The onset of labor does not have to be avoided because the effects on fetal wellbeing of contractions and hormones distributed while birth is initiated are well documented [27,28], even if a CS should become necessary in the process.

In vaginally completed breech deliveries, we were able to show that a high BW/CVO ratio is associated with significantly longer birth duration (414 min in vaginal low rate group, vLR versus 557 min in vaginal high rate group, vHR, *p* = 0.001, Table 4), accompanied by higher rates of epidural anesthesia in the vHR group (Table 4). This is in line with our hypothesis that a high fetal birth weight in combination with a short CVO leads to a hindered movement of the fetus through the birth canal, which is also explained by higher cesarean section rates (Table 1). Of note, fetal morbidity was not significantly different in our analysis (Table 4). Interestingly, manual assistance rates were not significantly different between vHR and vLR. Logistic regression analyzes revealed a positive correlation of the BW/CVO ratio and assisted arm delivery (‘Louwen maneuver’) rates (Table 5). The Louwen maneuver has been described by Louwen et al. [6]. Birth weight alone correlated also positively with assisted arm delivery rates but CVO on its own did not (Table 5). These data suggest that the BW/CVO ratio is predictive for the necessity to perform manual assistance during vaginal birth but fetal birth weight might have greater impact. Settings in which fetal birth weight and the CVO are immensely disadvantageous rather result in a cesarean section than in complicated vaginal deliveries.

A strength and novelty of this study is the analysis of the combined impact of fetal birth weight and pelvimetric measurements with clear results on how both parameters greatly influence birth course in primiparous women with a fetus in breech presentation. MRI assessment of the pelvis might not be feasible for most obstetric centers but the obstetric conjugate is also measurable by clinical examination. Values and the BW/CVO ratio might be used in women’s counselling, thus enabling an evidence based shared decision on the attempted birth mode when a breech presentation occurs at term. Since Klemt et al. published that a CVO of beneath 12 cm is correlated with a high cesarean section rate [11], many patients in our center with a narrow CVO opted for an elective cesarean section. This might lead to a systemic bias in our cohort because fewer patients with CVO values of beneath 12 cm could have been included in our study. Another limitation of our study is that birth weight and CVO are measured with different methodology (weighbridge and MRI). Producing a ratio of two differently acquired values is highly artificial. Thus, the BW/CVO ratio can be used to show correlations but translation into clinical practice might be difficult to reproduce.

## 5. Conclusions

In this study we were able to show that a high fetal weight in combination with a low CVO is predictive for cesarean section, results in longer birth duration and correlates with the necessity to perform manual assistance of the arm delivery in primiparous women at term giving birth to a fetus in breech presentation. Fetal morbidity is not impacted by the BW/CVO ratio. This study gives valuable input and highly important evidence for patients counseling in breech cases at term. Fetal birth weight and pelvic measurements and their combined impact on birth outcome should be discussed with patients in order to enable their evidence-based decision on delivery mode attempt.

## Figures and Tables

**Table 1:** Vaginally intended deliveries–Outcom in low ratio Group (LR) and high ratio group (HR).

Characteristic	Lr BW/CVO < 257.8 kg/cm *n* = 374	HR BW/CVO ≥ 257.8 kg/cm *n* = 374	*p* Value
Age (mean, standard deviation (SD); year (y))	31.4 (±3.8)	31.2 (±4.1)	0.795
BMI (mean, SD; kg/m2)	22.6 (±3.5)	23.3 (±3.6)	0.001
Duration of pregnancy (mean, SD; days)	277 (±8)	282 (±7)	<0.001
Maternal preconditions	57 (15.2%)	43 (11.5%)	0.133
Birth weight (mean, SD; gram)	3064.7 (±261)	3613.1 (±339)	<0.001
Obstetric conjugate (mean, SD; cm)	13.14 (0.87)	12.65 (0.82)	<0.001
Cesarean section rate (n, %)	106 (28.3%)	188 (50.3%)	<0.001
Epidural anesthesia	232 (62.0%)	259 (69.3%)	0.038
Arterial umbilical chord pH < 7	4 (1.08%)	1 (0.27%)	0.177
5 min APGAR < 4	2 (0.6%)	3 (0.4%)	0.661
NICU > 4 days	19 (5.1%)	(27 7.2%)	0.223
Intubation > 24 h	7 (1.9%)	3 (0.8%)	0.203
Neurological deficit	2 (0.5%)	2 (0.5%)	>0.99
Birth injury of the newborn	3 (0.8%)	4 (1.1%)	0.704
Newborn Infection	18 (4.8%)	26 (7.0%)	0.214
Congenital illness	10 (2.7%)	9 (2.4%)	0.816
PREMODA Score	21 (5.6%)	28 (7.5%)	0.301
PREMODA Score possibly related to birth mode	8 (2.1%)	13 (3.5%)	0.268

**Table 2:** Vaginally intended deliveries (*n* = 758), logistic regression analysis of CS rate.

Variable 1	Variable 2	r^2^	*p* Value	Inverted Prediction CS-Rate = 0.3	Inverted Prediction CS Rate = 0.5
CS rate	BW/CVO ratio	0.065	<0.001	240.0 g/cm	283.8 g/cm
CS rate	Birth weight	0.047	<0.001	3039 g	3691 g
CS rate	CVO	0.007	0.012	14.7 cm	11.0 cm

**Table 3:** Cesarean sections, indications (*n* = 294).

Characteristic	cLR BW/CVO < 257.8 kg/cm *n* = 106	cHR BW/CVO ≥ 257.8 kg/cm *n* = 188	*p* Value
Mother’s wish	5 (4.7%)	9 (4.8%)	0.978
Birth arrest in stage I	38 (35.9%)	79 (42.2%)	0.299
Birth arrest in stage II	32 (30.2%)	53 (28.2%)	0.717
Non-reassuring fetal heart rate	41 (38.7%)	40 (21.3%)	0.001
Prolapse of umbilical cord	4 (3.8%)	5 (2.7%)	0.594
Feto-pelvical disproportion	2 (1.9%)	17 (9.0%)	0.017
Suspected amniotic infection	4 (3.8%)	3 (1.6%)	0.240

**Table 4:** Vaginal deliveries in an upright position–Outcome (*n* = 366).

Characteristic	vLR BW/CVO < 257.8 kg/cm *n* = 218	vHR BW/CVO ≥ 257.8 kg/cm *n* = 148	*p* Value
Epidural anesthesia	124 (56.9%)	100 (67.6%)	0.040
Manual assistance required	91 (41.7%)	73 (49.3%)	0.152
Manually assisted head delivery (‘Frank Nudge’)	84 (38.5%)	69 (46.6%)	0.124
Manually assisted arm delivery (‘Louwen maneuver’)	39 (17.9%)	37 (25.0%)	0.100
Duration of Birth (mean, SD; minutes)	414 (±260)	557 (±331)	<0.001
PREMODA Score	7 (3.2%)	10 (6.8%)	0.114
PREMODA Score possibly related to birth mode	2 (0.9%)	5 (3.4%)	0.092
Non-perineal injuries (vaginal tear, labial or clitoral tear)	85 (39.0%)	67 (45.3%)	0.232
Perineal injury	126 (58.6%)	79 (54.9%)	0.482
III° and IV° Perineal injury	4 (1.8%)	6 (4.1%)	0.201

**Table 5:** Vaginal deliveries in an upright position–logistic regression analasis of manual assistance (*n* = 366).

Variable 1	Variable 2	r^2^	*p* Value
Manual assistance	Birth weight/CVO	0.0042	0.146
Manual assistance	Birth weight	0.0045	0.135
Manual assistance	CVO	0.0000	0.989
Assisted head delivery	Birth weight/CVO	0.0044	0.141
Assisted head delivery	Birth weight	0.0056	0.095
Assisted head delivery	CVO	0.0002	0.766
Assisted arm delivery	Birth weight/CVO	0.0147	0.020
Assisted arm delivery	Birth weight	0.0215	0.005
Assisted arm delivery	CVO	0.002	0.386

## Data Availability

Not applicable.

## Supplementary Materials

The following supporting information can be downloaded at: https://www.mdpi.com/article/10.3390/jcm11113201/s1, Table S1: Vaginally intended deliveries–demographic data of the whole study cohort (*n* = 758).
