# Supplementary material for: Combined Assessment of the Obstetrical Conjugate and Fetal Birth Weight Predicts Birth Mode Outcome in Vaginally Intended Breech Deliveries of Primiparous Women—A Frabat Study
Source: J Clin Med. 2022 Jun 3;11(11):3201. doi: 10.3390/jcm11113201 (PMC9181300; doi:10.3390/jcm11113201)
Supplement: Supplementary file 1 [file jcm-11-03201-s001.zip › jcm-1719059-supplementary.pdf]

Supplementary Materials

Table S1. Vaginally intended deliveries–demographic data of the whole study cohort (*n* = 758).

**Characteristic; n = 748 (whole cohort)**	
**Age (years; mean, SD)**	31.3 (± 3.9)
**BMI (kg/m², mean, SD)**	23.0 (± 3.6)
**Duration of pregnancy (days; mean, SD)**	280 (± 7.9)
**Maternal preconditions count**	100 (13.4%)
**Birth weight (gram; mean, SD)**	3339 (± 408)
**Obstetric conjugate (cm; mean, SD)**	12.9 (± 0.83)
**Cesarean section**	293 (39.2%)
